# Mathematical Model Explaining the Role of CDC6 in the Diauxic Growth of CDK1 Activity during the M-Phase of the Cell Cycle

**DOI:** 10.3390/cells8121537

**Published:** 2019-11-28

**Authors:** Mateusz Dębowski, Zuzanna Szymańska, Jacek Z. Kubiak, Mirosław Lachowicz

**Affiliations:** 1 Faculty of Mathematics, Informatics and Mechanics, Institute of Applied Mathematics and Mechanics, University of Warsaw, ul. Banacha 2, 02-097 Warsaw, Poland; 2 Institute of Mathematics, Polish Academy of Sciences, ul. Śniadeckich 8, 00-656 Warsaw, Poland or; 3 ICM, University of Warsaw, ul. Tyniecka 15/17, 02-630 Warsaw, Poland; 4 CNRS, Institute of Genetics and Development of Rennes, Univ Rennes, UMR 6290, Cell Cycle Group, Faculty of Medicine, F-35000 Rennes, France; 5 Laboratory of Regenerative Medicine and Cell Biology, Military Institute of Hygiene and Epidemiology (WIHE), ul. Kozielska 4, 01-163 Warsaw, Poland

**Keywords:** cell cycle, M-phase entry, mathematical model, dynamical system, diauxic dynamics, CDC 6, CDK 1, *Xenopus laevis* embryo

## Abstract

In this paper we propose a role for the CDC6 protein in the entry of cells into mitosis. This has not been considered in the literature so far. Recent experiments suggest that CDC6, upon entry into mitosis, inhibits the appearance of active CDK1 and cyclin B complexes. This paper proposes a mathematical model which incorporates the dynamics of kinase CDK1, its regulatory protein cyclin B, the regulatory phosphatase CDC25 and the inhibitor CDC6 known to be involved in the regulation of active CDK1 and cyclin B complexes. The experimental data lead us to formulate a new hypothesis that CDC6 slows down the activation of inactive complexes of CDK1 and cyclin B upon mitotic entry. Our mathematical model, based on mass action kinetics, provides a possible explanation for the experimental data. We claim that the dynamics of active complexes CDK1 and cyclin B have a similar nature to diauxic dynamics introduced by Monod in 1949. In mathematical terms we state it as the existence of more than one inflection point of the curve defining the dynamics of the complexes.

## 1. Introduction

The mitotic cell cycle is an ordered sequence of events, grouped into four phases: $G_{1}$, S, $G_{2}$ and M, during which the eukaryotic cell doubles its content, and divides into two daughter cells. The classical models for studies of cell cycle molecular machinery are oocytes and early embryos. These have the distinguishing property of being transcriptionally silent. This implies that the molecular machinery governing oocyte maturation and early embryo development is based on the maternal information accumulated during oocyte growth. While many different proteins regulate the progression of the cell cycle and the transitions between cell cycle phases, the major regulatory mechanisms are based on similar processes in all phases. Two main classes of proteins involved in cell cycle control are cyclins and enzymes called cyclin dependent kinases—CDKs. During individual phases a specific cyclin accumulates in the cell, associates with an appropriate kinase and with the help of other enzymes activates the kinase/cyclin complex. The appropriate level of an active complex triggers the transition to the next phase of the cell cycle.

A protein complex responsible for the transition from $G_{2}$ to M is formed from CDK1 kinase and cyclin B, the latter being abbreviated as CYCB [3,4]. The complex of CDK1 and CYCB, denoted by CDK1/CYCB, can be in one of two states, inactive or active, abbreviated as $\mathrm{CDK}1/{\mathrm{CYC}\;B}_{N}$ and $\mathrm{CDK}1/{\mathrm{CYC}\;B}_{A}$, respectively [3]. $\mathrm{CDK}1/{\mathrm{CYC}\;B}_{N}$ is dephosphorylated by active CDC25 phosphatase, denoted by ${\mathrm{CDC}25}_{A}$ (inactive phosphatase CDC25 is denoted by ${\mathrm{CDC}25}_{N}$). ${\mathrm{CDC}25}_{A}$ has the ability to pull away the phosphoryl group from two amino acids Tyr15 and Thr14 of the $\mathrm{CDK}1/{\mathrm{CYC}\;B}_{N}$ complex, making it active. This dephosphorylation results in the creation of $\mathrm{CDK}1/{\mathrm{CYC}\;B}_{A}$. $\mathrm{CDK}1/{\mathrm{CYC}\;B}_{A}$ induces a cascade of phosphorylation of numerous substrates that change the character of cellular proteins from interphase to mitotic. These changes—necessary for mitotic progression—modify structures such as the cytoskeleton, membranes and DNA (condensation). Moreover, activation of CDC25 phosphatase occurs due to its interaction with active $\mathrm{CDK}1/{\mathrm{CYC}\;B}_{A}$ complexes resulting in very powerful positive feedback between CDK1 and CDC25 that governs the CDK1 activation upon the entry into M-phase.

Summarising, a subtle equilibrium between CDC25 and CDK1 enzymes is maintained at the beginning of the $G_{2}$ to M-phase transition process. The association of CDK1 molecules with constantly synthesised CYCB results in the formation and accumulation of $\mathrm{CDK}1/{\mathrm{CYC}\;B}_{N}$. This complex is inactive because it is phosphorylated on Tyr15/Thr14 since CDC25 remains inactive. It was believed for a long time that, at some point, a spontaneous activation of the first molecules of CDK1/CYCB triggers a positive feedback between CDK1 and CDC25. Active molecules of CDK1/CYCB start CDC25 activation. Active CDC25, denoted as ${\mathrm{CDC}25}_{A}$, activates new molecules of $\mathrm{CDK}1/{\mathrm{CYC}\;B}_{N}$. This in turn triggers a dramatic acceleration of biochemical events leading to full activation of the whole pool of CDK1/CYCB complexes present in the cell. Moreover, recently Vigneron et al. [5] have shown that another complex containing CDK1, namely, CDK1/CYCA, triggers the activation of CDK1/CYCB.

A number of mathematical models have been proposed to describe and understand mitotic cell cycle progression. For instance, some have used the stochastic approach to capture non-deterministic aspects of the process and emphasised that noise is an important factor influencing cell dynamics [6,7,8,9,10,11,12]. The common approach, however, is the deterministic one that usually is based on systems of ordinary differential equations. Some authors considered large systems of that type in order to investigate transitions between cell cycle phases. Such systems describing the dynamics of different proteins and enzymes were usually analysed only numerically, e.g., [13,14,15,16,17,18,19,20]. Some models have considered the activation of CDK1, e.g., [21,22,23]. There are cell cycle models based not only on standard ordinary differential equations, but also on delay differential equations [24,25]. For example, Busenberg et al. [24] contains a rigorous analysis of the model. Interesting analytic results can be found in other papers [26,27,28,29,30]. We emphasise the analytic results obtained by Ferrell et al. who described the cell cycle using ordinary differential equations and proved the existence of oscillatory dynamics of the cell cycle, switch-like behaviour of activity of CDK1 and a bistability system [31,32].

The purpose of our work is to deepen the understanding of the cell cycle process. We are particularly focused on the $G_{2}$ to M-phase transition process and we aim at investigating the role of the CDC6 protein in entering into the M-phase of the cell cycle. CDC6, up to now, was known as an essential ATPase active in the S phase and responsible for the initiation of DNA replication.

Recent experiments made in *Xenopus laevis* one-cell embryo cell-free extract suggest that CDC6 has an important role in the delay of $G_{2}$ to M-phase transition [1]. This experimental system, however, simplified, as no nuclear DNA is present and all genetic information, both proteins and RNA, are of maternal origin and were accumulated in oocytes before the embryo development was triggered, allowed us to analyse the core molecular mechanisms of the cell cycle. We formulate a new hypothesis that explains this delay in terms of diauxic-like activation of CDK1/CYCB. In a different context, diauxic behaviour was introduced by Monod [2] in 1949. We propose a new mathematical model that captures this new hypothesis. We present the analysis and numerical simulations of this new model, suggesting how CDC6 regulates the dynamics of CDK1/CYCB activation upon M-phase entry.

**Figure 1:**
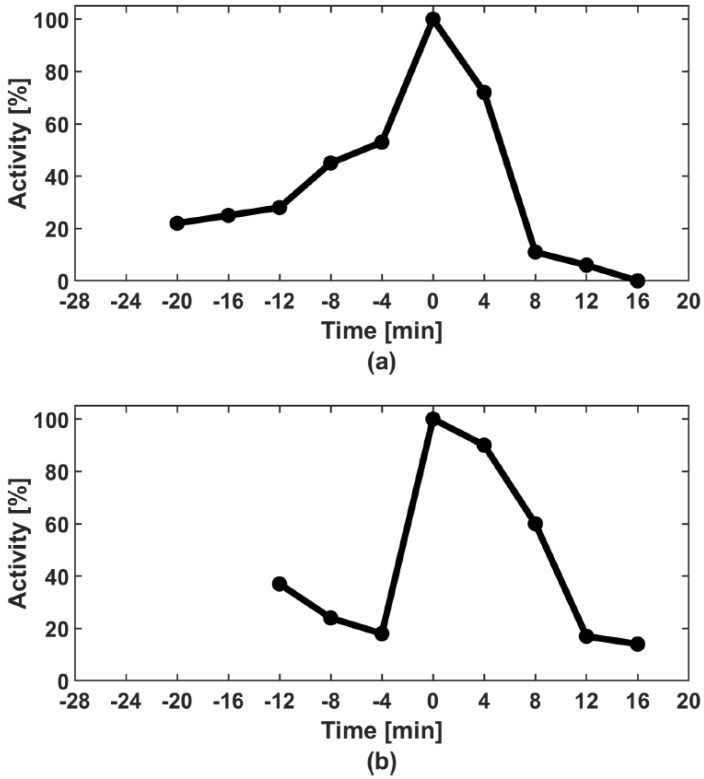
$\mathrm{CDK}1/{\mathrm{CYC}\;B}_{A}$ activity in the control extract containing physiological amounts of CDC6 (**a**) and in the extract immunodepleted of CDC6 (**b**). Note a slow and diauxic growth of $\mathrm{CDK}1/{\mathrm{CYC}\;B}_{A}$ activity in the control extract (**a**) and the very rapid activation in the absence of CDC6 (**b**). Curves reprinted from El Dika et al. [1].

**Figure 2:**
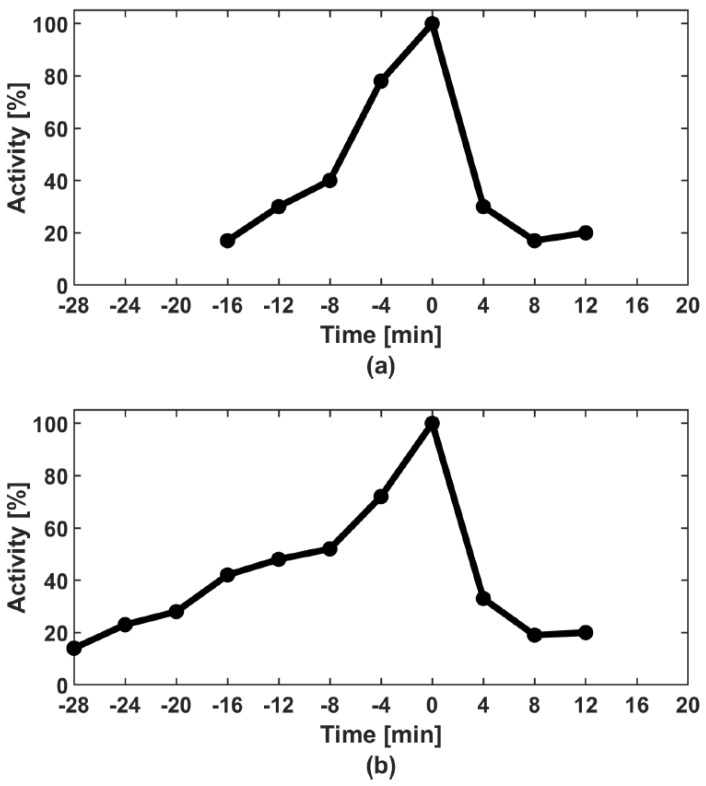
Differences in dynamics of $\mathrm{CDK}1/{\mathrm{CYC}\;B}_{A}$ activation curves in control extracts containing physiological amounts of CDC6. Two extreme examples are shown: Rapid activation taking 16 min (**a**) and slow activation taking 28 min (**b**). Note that the inflection points of the curves appear at different moments in relation to the maximum activity.

The structure of the paper is as follows. In Section 2 we present the material and methods used to obtain experimental results that inspired our subsequent research. In Section 3, at first, we introduce the biochemical model describing the basic events occurring when a cell enters mitosis. Then, we present the experimental results on the CDC6 protein that motivate our work. We show both the data reprinted from El Dika et al. [1] in Figure 1 and original results in Figure 2. We formulate a new hypothesis that captures the role of CDC6 in the process and the mathematical model corresponding to the biochemical one. Next, we present the numerical simulations of the proposed model and finally, Section 4 provides conclusions and directions for further research. In Appendix A we present the mathematical analysis of the presented model.

## 2. Material and Methods

### 2.1. Egg Collection and Activation

Spawn *Xenopus laevis* eggs were dejellied with 2% l-cysteine pH 7.81 in XB buffer (100 mM KCl, 1 mM MgCl_2_, 50 mM CaCl_2_, 10 mM HEPES and 50 mM sucrose pH 7.6). Next, they were washed in XB buffer, activated with 0.5 mg/mL calcium ionophore A23187 and extensively washed in XB.

### 2.2. Cell Free Extracts

Cytoplasmic extracts from calcium ionophore-activated one-cell embryos before the first embryonic mitosis were prepared according to El Dika et al. [1]. In short, embryos were cultured at 21 °C in XB buffer for 60–70 min postactivation, transferred into 5 mL ultraclear^TM^ centrifuge tubes (Beckman Coulter, Roissy, France) in 0.5 mL of XB buffer containing 0.1 mM AEBSF, a protease inhibitor, at 4°. They were subjected to three consecutive centrifugations: The first short spin to remove XB excess and pack the embryos, the second 10,000× *g* spin at 4 °C for 10 min to separate the cell-free fractions, and the final 10,000× *g* clarification spin of the supernatant at 4 °C for 10 min. The supernatant was then incubated at 21 °C. Aliquots were taken out every 4 min and stored at −80 °C.

### 2.3. CDK1 Activity Measurements

Samples of cell-free extracts were diluted in MPF buffer supplemented with: 0.5 mM sodium orthovanadate, 5 μg/μL of leupeptin, aprotinin, pepstatin and chymostatin, 0.4 mg/mL H1 histone (type III-S), 1μCi [γ32P] ATP (specific activity: 3000 Ci/mmol; Amersham Biosciences, UK) and 0.8 mM ATP. After incubation at 30 °C for 30 min, phosphorylation reactions were stopped by adding Laemmli sample buffer and heated at 85 °C for 5 min. Histone H1 was separated by SDS-PAGE and incorporated radioactivity was measured by autoradiography of the gel using a STORM phosphorimager (Amersham Biosciences, Buckinghamshire, UK) followed by data analysis with ImageQuant 5.2 software.

### 2.4. CDC6 Immunodepletion

Immunodepletion of CDC6 from egg extracts was carried out using AffiPrep Protein A beads (Sigma, USA) conjugated with the anti-CDC6 or with the preimmune serum overnight in 4 °C; 200 mL of beads were washed four times with XB buffer (pH 7.6) and incubated with 400 mL of extracts. After 30 min of incubation at 4 °C, extracts were centrifuged, beads were removed and supernatant was recovered. Two consecutive runs of immunodepletion were required to remove 90% of CDC6, as shown in El Dika et al. [1].

## 3. Results

### 3.1. Biochemical Model and the New Hypothesis

CYCB concentration gradually increases during the $G_{2}$ phase (cf. Equation (5)). CYCB pairs with protein kinase CDK1 creates an inactive (phosphorylated) complex—$\mathrm{CDK}1/{\mathrm{CYC}\;B}_{N}$ (cf. Equation (1)). Inactive complex $\mathrm{CDK}1/{\mathrm{CYC}\;B}_{N}$ upon its interaction with phosphatase ${\mathrm{CDC}25}_{A}$ becomes activated, thus the concentration of active complexes $\mathrm{CDK}1/{\mathrm{CYC}\;B}_{A}$ increases (cf. Equation (2)). Conversely, complex $\mathrm{CDK}1/{\mathrm{CYC}\;B}_{A}$ activates phosphatase ${\mathrm{CDC}25}_{N}$ causing the appearance of more ${\mathrm{CDC}25}_{A}$ (cf. Equation (3)). Summarising, ${\mathrm{CDC}25}_{A}$ and $\mathrm{CDK}1/{\mathrm{CYC}\;B}_{A}$ form a positive feedback loop. The M phase begins when the concentration of active $\mathrm{CDK}1/{\mathrm{CYC}\;B}_{A}$ exceeds the threshold value.

Recent experimental studies provoke intriguing questions about the role of the CDC6 protein in slowing down the activation of $\mathrm{CDK}1/{\mathrm{CYC}\;B}_{N}$ complexes. Figure 1 shows the concentration of $\mathrm{CDK}1/{\mathrm{CYC}\;B}_{A}$ (from a biochemical point of view, simply the CDK1 activity) obtained on the basis of molecular experiments in two cases: (a) With CDC6; and (b) without CDC6 (after removal of CDC6 from the experimental system) [1]. In the experimental setting with CDC6, one can notice a slower increase in the concentration of $\mathrm{CDK}1/{\mathrm{CYC}\;B}_{A}$. Therefore, in the experimental system with CDC6 the entry into mitosis is delayed. Our main goal is to explain the role of CDC6 in the observed phenomenon.

**Figure 3:**
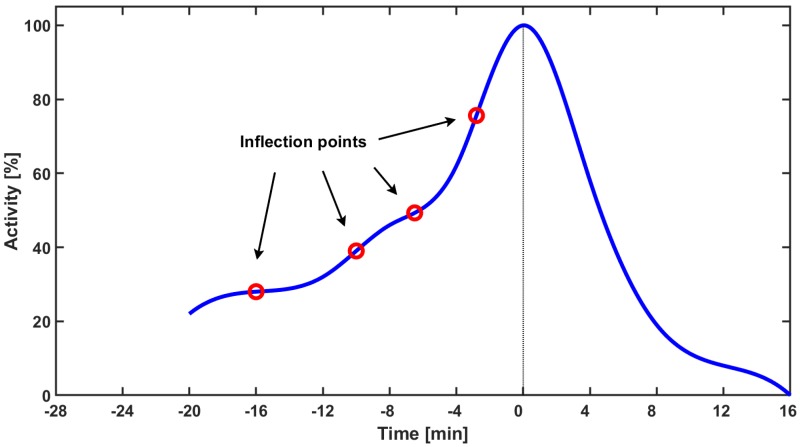
The smoothed curve obtained on the basis of experimental data presented in Figure 1a. Red circles indicate approximate location of inflection points for the setting with CDC6 upon M-phase entry.

**Figure 4:**
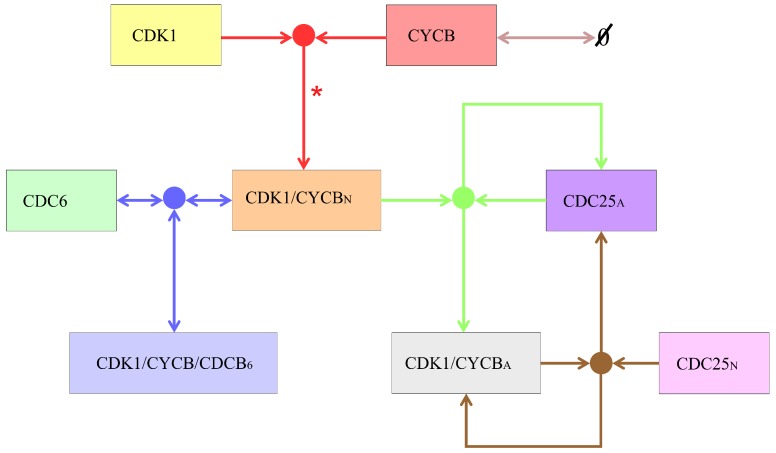
The schematic diagram of the considered system. Colours of arrows and dots correspond to colours of Equations (1)–(5). For simplicity we do not consider the potential marginal separation of the complex $\mathrm{CDK}1/{\mathrm{CYC}\;B}_{N}$ into CDK1 and CYCB. On the diagram we indicate this by “*”.

The diauxic growth of CDK1 activity was clearly noticed in previous studies of *Xenopus laevis* one-cell embryo cell-free extracts ([33]: Figure 1A bottom, Figure 2A right, Figure 3A right, [34]: Figure 2A bottom and [28]: Figure 1V). Furthermore, in our own research, we always observed the same type of behaviour of CDK1 ([35]: Figures 1A, 2A, 3A and 6A, [36]: Figures 2A, 3A, 6A and 7A,B and [37]: Figures 6A and 7B). Moreover, the diauxic growth of CDK1 activity is not an artefact due to the cell-free system because it was also observed in individual *Xenopus laevis* one-cell embryos ([38]: Figure 1A); however, it is more clear in the vegetal hemisphere where the CDK1 activation is delayed and proceeds with lower dynamics ([38]: Figure 1B). The precise dynamics of the diauxic growth of CDK1, i.e., inflection times and slope of the curve, varies form one experiment to another. For this reason, the average curves showing the dynamics of CDK1 activation upon the M-phase entry in *Xenopus laevis* one-cell embryos do not preserve the diauxic character ([35]: Figure 4B), where the average curve of 16 independent experiments does not show any inflection points. In Figure 2 in the current paper we show two examples of the fast and slow growth of CDK1 activity in two independent experiments illustrating this problem well. The average curve of these two experiments also does not show the inflection points clearly visible in each experimental curve (data not shown).

We hypothesise that CDC6 binds to $\mathrm{CDK}1/{\mathrm{CYC}\;B}_{N}$ and creates a new CDK1/CYCB/CDC6 complex, preventing $\mathrm{CDK}1/{\mathrm{CYC}\;B}_{N}$ from being activated by ${\mathrm{CDC}25}_{A}$ phosphatase (cf. Equation (4)). The resulting CDK1/CYCB/CDC6 complexes constantly break down into CDC6 and $\mathrm{CDK}1/{\mathrm{CYC}\;B}_{N}$ that constantly associate again. The more $\mathrm{CDK}1/{\mathrm{CYC}\;B}_{N}$ accumulate in the cell, the more $\mathrm{CDK}1/{\mathrm{CYC}\;B}_{N}$ complexes are activated by residual ${\mathrm{CDC}25}_{A}$. The formation of CDK1/CYCB/CDC6 prolongs a very slow increase in the appearance of $\mathrm{CDK}1/{\mathrm{CYC}\;B}_{A}$ complexes. A slowdown in $\mathrm{CDK}1/{\mathrm{CYC}\;B}_{A}$ increase is visible as the flattening of the curve in Figure 1a. The experimental data leads to a new hypothesis on the mutual interaction between CDC6 and $\mathrm{CDK}1/{\mathrm{CYC}\;B}_{N}$, which determines the dynamics of $\mathrm{CDK}1/{\mathrm{CYC}\;B}_{A}$ upon mitotic entry. Our mathematical model, based on the law of mass action, bolsters this hypothesis. We suggest that the dynamics of $\mathrm{CDK}1/{\mathrm{CYC}\;B}_{A}$ are similar to diauxic dynamics introduced by Monod [2]. In mathematical terms, we state it as the existence of more than one inflection point on the curve defining the dynamics of the complexes, cf. Figure 3. Indeed, in the present model, we observe three or four inflexion points.

The second part of our hypothesis is that the reaction speed of $\mathrm{CDK}1/{\mathrm{CYC}\;B}_{N}$ and CDC6 binding depends on active $\mathrm{CDK}1/{\mathrm{CYC}\;B}_{A}$ in a switch like mode. This means that when the concentration of $\mathrm{CDK}1/{\mathrm{CYC}\;B}_{A}$ is less than the concentration value, then the reaction speed of CDK1/CYCB/CDC6 formation is low. When the $\mathrm{CDK}1/{\mathrm{CYC}\;B}_{A}$ is higher than the threshold value the reaction speed becomes much faster resulting in a two-step CDK1 activation visible in biological experiments as an inflection of the activation curve of CDK1. Experimental data show clearly that this activation depends on the presence of CDC6 [1].

Summarising, we consider the biochemical model that takes into account eight species, the descriptions of which are provided in Table 1, whereas the scheme of their mutual interactions is provided in Figure 4.

We consider the following five reactions (i.e., Equations (1)–(5)).

(1) $$\mathrm{CDK}1+\mathrm{CYC}\;B\overset{\;\alpha_{1}\;}{\to }\mathrm{CDK}1/{\mathrm{CYC}\;B}_{N},$$

(2) $$\mathrm{CDK}1/{\mathrm{CYC}\;B}_{N}+{\mathrm{CDC}25}_{A}\overset{\;\alpha_{2}\;}{\to }\mathrm{CDK}1/{\mathrm{CYC}\;B}_{A}+{\mathrm{CDC}25}_{A},$$

(3) $$\mathrm{CDK}1/{\mathrm{CYC}\;B}_{A}+{\mathrm{CDC}25}_{N}\overset{\;\alpha_{3}\;}{\to }\mathrm{CDK}1/{\mathrm{CYC}\;B}_{A}+{\mathrm{CDC}25}_{A},$$

(4) $$\mathrm{hyp}:\;\mathrm{CDK}1/{\mathrm{CYC}\;B}_{N}+\mathrm{CDC}6\overset{\alpha_{4}\cdot f\left(\mathrm{CDK}1/{\mathrm{CYC}\;B}_{A}\right)}{\underset{\delta }{⇌}}\mathrm{CDK}1/\mathrm{CYC}\;B/\mathrm{CDC}6,$$

(5) $$\emptyset \overset{\beta \cdot K_{cycB}}{\underset{\beta }{⇌}}\mathrm{CYC}\;B$$

We want to emphasise that Equations (1)–(3) and (5) correspond to the current state of knowledge. Equation (4) reflects the new hypothesis and is our contribution to understanding the phenomenon. In summary, taking Equation (4) into account is the first part of the new hypothesis. The speed of Equation (4) described by the function *f* is the second part of the hypothesis.

### 3.2. Mathematical Model

Assuming mass action kinetics for Equations (1)–(5) we transform the biochemical model into the system of eight ordinary differential equations (ODEs) with the following notation

$$\begin{array}{c} x=\mathrm{CDK}1\;,\;x_{a}=\mathrm{CDK}1/{\mathrm{CYC}\;B}_{A}\;,\;x_{n}=\mathrm{CDK}1/{\mathrm{CYC}\;B}_{N}\;,\;y_{a}={\mathrm{CDC}25}_{A}\;,\\ y_{n}={\mathrm{CDC}25}_{N}\;,\;z=\mathrm{CDC}6\;,\;w=\mathrm{CDK}1/\mathrm{CYC}\;B/\mathrm{CDC}6\;,\;c=\mathrm{CYC}\;B\;. \end{array}$$

We have

(6) $$\begin{array}{ccc} \dot{x} & = & -\alpha_{1}xc\;,\\ {\dot{x}}_{a} & = & \alpha_{2}x_{n}y_{a}\;,\\ {\dot{x}}_{n} & = & \alpha_{1}xc-\alpha_{2}x_{n}y_{a}-\alpha_{4}f\left(x_{a}\right)x_{n}z+\delta w\;,\\ {\dot{y}}_{a} & = & \alpha_{3}x_{a}y_{n}\;,\\ {\dot{y}}_{n} & = & -\alpha_{3}x_{a}y_{n}\;,\\ \dot{z} & = & -\alpha_{4}f\left(x_{a}\right)x_{n}z+\delta w\;,\\ \dot{w} & = & \alpha_{4}f\left(x_{a}\right)x_{n}z-\delta w\;,\\ \dot{c} & = & -\alpha_{1}xc+\beta \left(K_{\mathrm{CYC}\;B}-c\right)\;, \end{array}$$

where $\alpha_{1},\alpha_{2},\alpha_{3},\alpha_{4},\beta ,\delta$ are positive parameters and $f\left(x\right)=\omega +\frac{\nu x^{k}}{v_{th}^{k}+x^{k}}$. The function *f* is the Hill function that describes switch-like behaviour, where ν is a positive coefficient, *k* is a Hill coefficient, $v_{th}$ is the threshold value of the switch and ω is the basic rate when $x_{a}=0$. The function *f* describes the reaction rate of $\mathrm{CDK}1/{\mathrm{CYC}\;B}_{N}$ associated with CDC6 resulting in the formation of CDK1/CYCB/CDC6 complexes (cf. Equation (4)). In the system of ordinary differential equations, Equation (6) appears in the 7th equation describing the dynamics of CDK1/CYCB/CDC6 and, due to the law of mass action, in the 3rd and 6th equations describing the dynamics of $\mathrm{CDK}1/{\mathrm{CYC}\;B}_{N}$ and CDC6, respectively. The process of CDK1/CYCB/CDC6 formation seems to be highly nonlinear and we assume its rate to be $\mathrm{CDK}1/{\mathrm{CYC}\;B}_{A}$ dependent. There exists a similar mechanism governing interactions between CDK1 and CDC6 in S-phase. If $\mathrm{CDK}1/{\mathrm{CYC}\;B}_{A}$ is low then the majority of CDC6 is not phosphorylated. However, with an increase of $\mathrm{CDK}1/{\mathrm{CYC}\;B}_{A}$ more phosphorylated CDC6 appears in the cell [39]. The function *f* is bounded as it plays the role of a rate coefficient. The typical way of modelling such a nonlinear dependence is based on the Hill function, see, e.g., [40].

Taking into consideration the biological constraints, we propose the following initial data

(7) $$\begin{array}{ccc} x(0) & = & K_{\mathrm{CDK}1}-\varepsilon_{x_{a}}-\varepsilon_{x_{n}}-\varepsilon_{w}>0\;,\\ x_{a}\left(0\right) & = & \varepsilon_{x_{a}}\ll K_{\mathrm{CDK}1}\;,\\ x_{n}\left(0\right) & = & \varepsilon_{x_{n}}\ll K_{\mathrm{CDK}1}\;,\\ y_{a}\left(0\right) & = & \varepsilon_{y_{a}}\ll K_{\mathrm{CDC}25}\;,\\ y_{n}\left(0\right) & = & K_{\mathrm{CDC}25}-\varepsilon_{y_{a}}\;,\\ z(0) & = & K_{\mathrm{CDC}6}-\varepsilon_{w}\;,\\ w(0) & = & \varepsilon_{w}\ll \min\left\{K_{\mathrm{CDK}1},K_{\mathrm{CDC}6}\right\}\;,\\ c(0) & = & 0\;. \end{array}$$

Equation (6) have the following conservation laws

(8) $$\begin{array}{c} x_{a}+x_{n}+x+w=K_{\mathrm{CDK}1}\;,\\ y_{a}+y_{n}=K_{\mathrm{CDC}25}\;,\\ z+w=K_{\mathrm{CDC}6}\;, \end{array}$$

where $K_{\mathrm{CDK}1},\;K_{\mathrm{CDC}25},\;K_{\mathrm{CDC}6}$ denote constants given at the initial time. In Appendix A we provide the mathematical analysis of the model.

We provide the standard non-dimensionalisation of Equation (6). In other words we relate all considered variables to their characteristic values. With the substitution

(9) $$\begin{array}{cccc} x^{*}=\frac{x}{K_{\mathrm{CDK}1}}\;, & x_{a}^{*}=\frac{x_{a}}{K_{\mathrm{CDK}1}}\;, & x_{n}^{*}=\frac{x_{n}}{K_{\mathrm{CDK}1}}\;, & w^{*}=\frac{w}{K_{\mathrm{CDK}1}}\;,\\ y_{a}^{*}=\frac{y_{a}}{K_{\mathrm{CDC}25}}\;, & y_{n}^{*}=\frac{y_{n}}{K_{\mathrm{CDC}25}}\;, & z^{*}=\frac{z}{K_{\mathrm{CDK}1}}\;, & c^{*}=\frac{c}{K_{\mathrm{CYC}\;B}}\;,\\ t^{*}=\beta \;t\;, & \gamma =\frac{K_{\mathrm{CDC}6}}{K_{\mathrm{CDK}1}}\;, & \nu_{th}^{*}=\frac{\nu_{th}}{K_{CDK1}}\;, & \delta^{*}=\frac{\delta }{\beta }\;,\\ \alpha_{1}^{*}=\frac{\alpha_{1}K_{\mathrm{CYC}\;B}}{\beta }\;, & \alpha_{2}^{*}=\frac{\alpha_{2}K_{\mathrm{CDC}25}}{\beta }\;, & \alpha_{3}^{*}=\frac{\alpha_{3}K_{\mathrm{CDK}1}}{\beta }\;, & \alpha_{4}^{*}=\frac{\alpha_{4}K_{\mathrm{CDK}1}}{\beta }\;,\\ \varepsilon_{x_{a}}^{*}=\frac{\varepsilon_{x_{a}}}{K_{\mathrm{CDK}1}}\;, & \varepsilon_{x_{n}}^{*}=\frac{\varepsilon_{x_{n}}}{K_{\mathrm{CDK}1}}\;, & \varepsilon_{y_{a}}^{*}=\frac{\varepsilon_{y_{a}}}{K_{\mathrm{CDC}25}}\;, & \varepsilon_{w}^{*}=\frac{\varepsilon_{w}}{K_{\mathrm{CDK}1}}\;, \end{array}$$

and omitting the stars for simplicity, we obtain

(10) $$\begin{array}{ccc} \dot{x} & = & -\alpha_{1}xc\;,\\ {\dot{x}}_{a} & = & \alpha_{2}x_{n}y_{a}\;,\\ {\dot{x}}_{n} & = & \alpha_{1}xc-\alpha_{2}x_{n}y_{a}-\alpha_{4}f\left(x_{a}\right)x_{n}z+\delta w\;,\\ {\dot{y}}_{a} & = & \alpha_{3}x_{a}y_{n}\;,\\ {\dot{y}}_{n} & = & -\alpha_{3}x_{a}y_{n}\;,\\ \dot{z} & = & -\alpha_{4}f\left(x_{a}\right)x_{n}z+\delta w\;,\\ \dot{w} & = & \alpha_{4}f\left(x_{a}\right)x_{n}z-\delta w\;,\\ \dot{c} & = & -\alpha_{1}xc+\left(1-c\right)\;. \end{array}$$

By Equation (8) it follows

(11) $$\begin{array}{c} x_{a}+x_{n}+x+w=1,\\ y_{a}+y_{n}=1,\\ z+w=\gamma \;. \end{array}$$

By Equation (7) we obtain

(12) $$\begin{array}{ccc} x(0) & = & 1-\varepsilon_{x_{a}}-\varepsilon_{x_{n}}-\varepsilon_{w}>0\;,\\ x_{a}\left(0\right) & = & \varepsilon_{x_{a}}\ll 1\;,\\ x_{n}\left(0\right) & = & \varepsilon_{x_{n}}\ll 1\;,\\ y_{a}\left(0\right) & = & \varepsilon_{y_{a}}\ll 1\;,\\ y_{n}\left(0\right) & = & 1-\varepsilon_{y_{a}}\;,\\ z(0) & = & \gamma -\varepsilon_{w}\;,\\ w(0) & = & \varepsilon_{w}\ll \min\left\{1,\gamma \right\}\;,\\ c(0) & = & 0\;. \end{array}$$

From the mathematical analysis presented in Appendix A we deduce that if the system contains even a small amount of $\mathrm{CDK}1/{\mathrm{CYC}\;B}_{A}$ or ${\mathrm{CDC}25}_{A}$ then $\mathrm{CDK}1/{\mathrm{CYC}\;B}_{A}$ and ${\mathrm{CDC}25}_{A}$ converge to full activation. This result is consistent with biological observations, because if the initial concentration of $\mathrm{CDK}1/{\mathrm{CYC}\;B}_{A}$ or ${\mathrm{CDC}25}_{A}$ is positive then the positive feedback loop starts and the biological system tends to its equilibrium state (called $S_{2}$) defined by the maximal concentrations of $\mathrm{CDK}1/{\mathrm{CYC}\;B}_{A}$ and ${\mathrm{CDC}25}_{A}$. If the initial concentrations of $\mathrm{CDK}1/{\mathrm{CYC}\;B}_{A}$ or ${\mathrm{CDC}25}_{A}$ are equal to zero, then the positive feedback loop does not start and the biological system tends to another equilibrium state (called $S_{1}$) defined by the concentrations of $\mathrm{CDK}1/{\mathrm{CYC}\;B}_{A}$ and ${\mathrm{CDC}25}_{A}$ equal to 0. Small perturbations of the initial concentrations from zero to positive values change the equilibrium points, and this is the biological reason for $S_{1}$ being unstable and $S_{2}$ being asymptotically stable.

We note that a further simplification of the reduced model, Equation (A3), considered in Appendix A is reasonable. For example taking x=0 and c=1 we may reduce this system to a system of three equations

(13) $$\begin{array}{ccc} {\dot{x}}_{a} & = & \alpha_{2}\left(1-\gamma +z-x_{a}\right)y_{a}\;,\\ {\dot{y}}_{a} & = & \alpha_{3}x_{a}\left(1-y_{a}\right)\;,\\ \dot{z} & = & -\alpha_{4}f\left(x_{a}\right)\left(1-\gamma +z-x_{a}\right)z+\delta \left(\gamma -z\right)\;. \end{array}$$

### 3.3. Numerical Simulations

**Figure 5:**
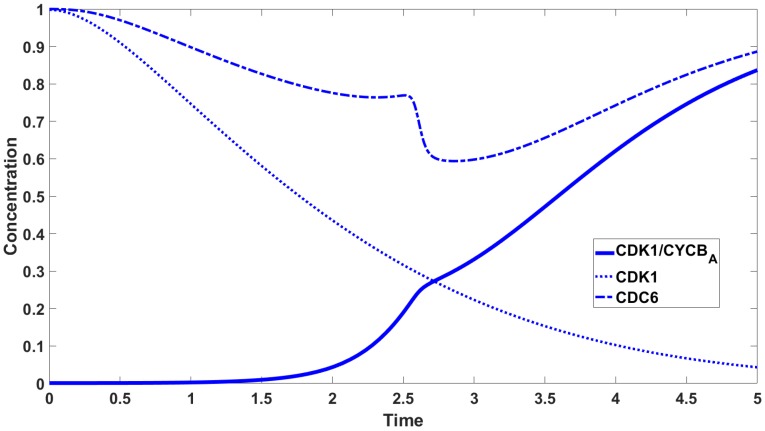
Concentration of CDK1, $\mathrm{CDK}1/{\mathrm{CYC}\;B}_{A}$, CDC6 in the presence of CDC6.

**Figure 6:**
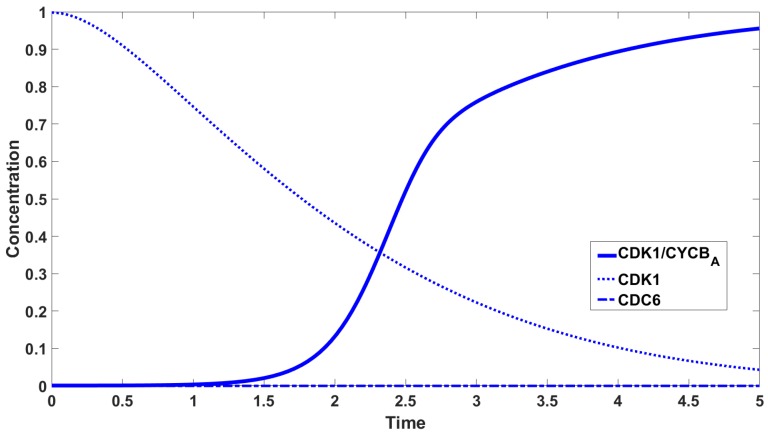
Concentration of CDK1, $\mathrm{CDK}1/{\mathrm{CYC}\;B}_{A}$, CDC6 in the absence of CDC6.

**Figure 7:**
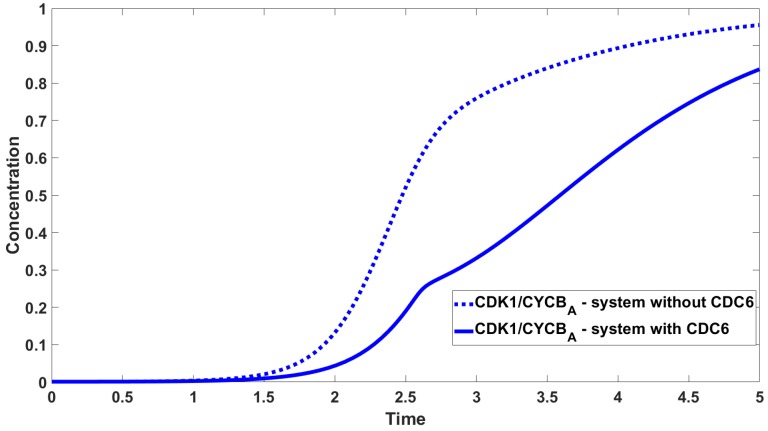
Comparison between concentration of $\mathrm{CDK}1/{\mathrm{CYC}\;B}_{A}$ in the absence and presence of CDC6. Solid line—system with CDC6; dotted line—system without CDC6.

**Figure 8:**
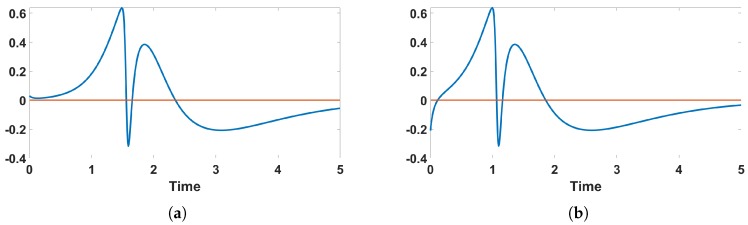
Graphs presenting second derivatives of $x_{a}$ showing the number of zeros, which indicates the number of inflection points. (**a**) corresponds to the case with the second derivative starting from a positive value and having three zeros. (**b**) corresponds to the case with the second derivative starting from a negative value and having four zeros.

To carry out the numerical simulations we use the Runge-Kutta 4th order method provided by Matlab. Parameters values used to carry out the numerical simulations are given in Table 2. Figure 5 shows the concentrations of CDK1, $\mathrm{CDK}1/{\mathrm{CYC}\;B}_{A}$, $\mathrm{CDK}1/{\mathrm{CYC}\;B}_{N}$, CDK1/CYCB/CDC6. The most interesting curve is $\mathrm{CDK}1/{\mathrm{CYC}\;B}_{A}$, where we observe three inflection points. The concentrations of species containing CDK1 are shown in Figure 6 with the concentration of CDC6 set to zero. Figure 7 shows the difference in activation: The timing and dynamics of the activation of $\mathrm{CDK}1/{\mathrm{CYC}\;B}_{A}$ in the presence and absence of CDC6. When CDC6 is present the activation has more than one inflection point, and mitosis starts later, whereas when CDC6 is absent, the activation is fast. In Figure 5 and Figure 7 we observe diauxic-type behaviour for the curve of $\mathrm{CDK}1/{\mathrm{CYC}\;B}_{A}$. According to our hypothesis, this is related to the mutual interaction between CDC6 and $\mathrm{CDK}1/{\mathrm{CYC}\;B}_{N}$. We link this kind of behaviour with the existence of multiple (three or four in this case) inflection points in the curve of $\mathrm{CDK}1/{\mathrm{CYC}\;B}_{A}$. The rigorous investigation of this fact leads to the analysis of behaviour of the second derivative of $\mathrm{CDK}1/{\mathrm{CYC}\;B}_{A}$ and more precisely its number of zeros. Figure 8 presents the graphs of the second derivative of $x_{a}$ obtained for the reduced system of three equations, Equation (13).

We may note that the numerical result given in Figure 8 has a rigorous nature as is visible by a careful estimation of the error of Matlab approximation. For example, considering Equation (13), we may provide (following the idea of [41]) the detailed analysis of the error

$$E_{n}=y_{n}-y\left(t_{n}\right)\;,$$

where $y(t_{n})$ is the value of the true solution at point $t_{n}$ and $y_{n}$ is the approximation of the solution at point $t_{n}$, showing that

$$|E_{n}|<4.5\cdot 10^{10}\cdot h^{4}\;.$$

Taking $h=\frac{1}{1000}$ the error is smaller than the variation in the second derivative. Moreover, the rounding error can be neglected because the machine epsilon (see [42]) is sufficiently small compared with *h*. The details of the estimation are not reported here. This leads to the conclusion that the result stating the number of zeros of the second derivative has a rigorous nature and there the number of inflection points of the variable $x_{a}$ is either three or four, which give diauxic behaviour.

## 4. Discussion

The proposed model captures the most important characteristics of the diauxic growth of CDK1 activation observed in biochemical experiments. Based on our previous experimental results [1] we claim that CDC6 is the most important factor which causes the inflection of the CDK1 activation curve. We have shown for the first time that CDC6 is an inhibitory protein acting on CDK1 during M-phase. Making use of our modelling setting, we hypothesise that CDC6 binds to $\mathrm{CDK}1/{\mathrm{CYC}\;B}_{N}$ forming CDK1/CYCB/CDC6. CDK1/CYCB/CDC6 formation results in slower activation of CDK1 and consequently a delayed entry into M-phase. From a biochemical perspective our results, both experimental and modelling, are particularly interesting because the inhibitory effect of CDC6 on CDK1 activation during M-phase was not shown previously.

The second part of our hypothesis stands for the switch-like dependence of the reaction rate of $\mathrm{CDK}1/{\mathrm{CYC}\;B}_{N}$ binding to CDC6 resulting in the formation of CDK1/CYCB/CDC6 (Equation (4)). Our assumption that the mentioned reaction rate depends on $\mathrm{CDK}1/{\mathrm{CYC}\;B}_{A}$ provides a good qualitative explanation of the observed diauxic dynamics of CDK1 activation. Further biological research is needed to investigate what molecular modification is necessary for this switch-like pattern of CDK1 activation. We can postulate that CDK1, at some threshold, phosphorylates CDC6 triggering the abrupt increase in CDC6 affinity to $\mathrm{CDK}1/{\mathrm{CYC}\;B}_{N}$. From a more general perspective, the slow rate of CDK1 activation is very likely important for the physiological course of mitotic processes such as chromatin condensation or spindle formation in such a large cell as the *Xenopus laevis* one-cell embryo.

One of the main goals of the paper was to describe mathematically the diauxic behaviour of CDK1 activation in the presence of CDC6 protein. However, this kind of approach has a wide spectrum of use and may be applied to a large variety of problems. Usually such complex biological systems are very difficult to treat rigorously from a mathematical point of view. The analysis of the error gives a chance for a rigorous statement based on the numerical simulations. We leave the details of this approach for a forthcoming paper.

Our results may affect the understanding of the process of cancerogenesis since CDC6 and its interactions with CDK1 play an important role in mitotic regulation and in cancer etiology [43,44]. The CDC6 role in M-phase regulation is not limited only to the mitotic cell cycle as shown in the current paper and in El Dika et al. [1], but also to the meiotic regulation in oocytes [45,46,47,48]. The requirement of CDC6 for the meiotic spindle formation in mice and *Xenopus laevis* oocytes suggests that it can also be involved in mitotic spindle formation. The diauxic growth of CDK1 activity determined by CDC6 may be in relation to the proper dynamics of spindle assembly not only through the fine tuning of microtubule dynamics, but also by the proper coordination with other players like actin filaments [49,50].

## Figures and Tables

**Table 1:** Species of the biochemical model involved in Equations (1)–(5).

Species	Description
CDK1	cyclin-dependent kinase 1
CYCB	cyclin B
$\mathrm{CDK}1/{\mathrm{CYC}\;B}_{A}$	active complex of CDK and CYCB
$\mathrm{CDK}1/{\mathrm{CYC}\;B}_{N}$	inactive complex of CDK and CYCB
${\mathrm{CDC}25}_{A}$	active phosphatase CDC25
${\mathrm{CDC}25}_{N}$	inactive phosphatase CDC25
CDC6	cell division cycle 6 ATPase
CDK1/CYCB/CDC6	complex of $\mathrm{CDK}1/{\mathrm{CYC}\;B}_{N}$ and CDC6

**Table 2:** Values of parameters used in simulations.

Parameter	Value	Parameter	Value	Parameter	Value
$\alpha_{1}$	1	δ	4	$\varepsilon_{x_{a}}$	0.001
$\alpha_{2}$	30	k	20	$\varepsilon_{x_{n}}$	0.001
$\alpha_{3}$	1	ν	8	$\varepsilon_{y_{a}}$	0
$\alpha_{4}$	7	$\nu_{th}$	0.25	$\varepsilon_{w}$	0
γ	1	ω	0.6		

## Appendix A. Mathematical Analysis of the Model

The global existence, uniqueness, positiveness and boundedness of the solutions follow directly from the form of Equation (10). Keeping in mind Equation (12), we obtain

(A1) $$\begin{array}{c} x\left(t\right),\;x_{a}\left(t\right),\;x_{n}\left(t\right),\;y_{a}\left(t\right),\;y_{n}\left(t\right),\;c\left(t\right)\in \left[0,1\right],\\ z(t)\in [0,\gamma ],\;w(t)\in [0,\min(1,\gamma )], \end{array}$$

for all t>0. From Equation (11) we obtain

(A2) $$\begin{array}{c} x_{n}=1-x_{a}-x-w,\\ y_{n}=1-y_{a},\\ w=\gamma -z. \end{array}$$

Using Equation (A2) we reduce Equation (10) to

(A3) $$\begin{array}{ccc} \dot{x} & = & -\alpha_{1}xc,\\ {\dot{x}}_{a} & = & \alpha_{2}\left(1-\gamma +z-x_{a}-x\right)y_{a},\\ {\dot{y}}_{a} & = & \alpha_{3}x_{a}\left(1-y_{a}\right),\\ \dot{z} & = & -\alpha_{4}f\left(x_{a}\right)\left(1-\gamma +z-x_{a}-x\right)z+\delta \left(\gamma -z\right)\\ \dot{c} & = & -\alpha_{1}xc+\left(1-c\right). \end{array}$$

Referring to equilibrium points we formulate the following proposition.

**Proposition** **A1.**
*In the set ${\left[0,1\right]}^{3}\times \left[0,\gamma \right]\times \left[0,1\right]$ there exist only two equilibrium points of Equation (A3):*

(1) $S_{1}=\left(0,0,0,\frac{1}{2}\left(\gamma -1-\frac{\delta }{\alpha_{4}\omega }+\sqrt{{\left(1-\gamma +\frac{\delta }{\alpha_{4}\omega }\right)}^{2}+4\frac{\delta \gamma }{\alpha_{4}\omega }}\right),1\right)\;,$
(2) $S_{2}=\left(0,1,1,\gamma ,1\right)\;.$

**Proof.** Let the equilibrium point of Equation (A3) be denoted by $\left(\bar{x},{\bar{x}}_{a},{\bar{y}}_{a},\bar{z},\bar{c}\right)$. We have

(A4) $$\begin{array}{c} -\alpha_{1}\bar{x}\bar{c}=0,\\ \alpha_{2}\left(1-\gamma +\bar{z}-{\bar{x}}_{a}-\bar{x}\right){\bar{y}}_{a}=0,\\ \alpha_{3}{\bar{x}}_{a}\left(1-{\bar{y}}_{a}\right)=0,\\ -\alpha_{4}f\left({\bar{x}}_{a}\right)\left(1-\gamma +\bar{z}-{\bar{x}}_{a}-\bar{x}\right)\bar{z}+\delta \left(\gamma -\bar{z}\right)=0,\\ -\alpha_{1}\bar{x}\bar{c}+\left(1-\bar{c}\right)=0. \end{array}$$

From the fifth equation of Equation (A4) we obtain $\bar{c}\neq 0$, and from the first and fifth equations of Equation (A4) we obtain $\bar{x}=0,\;\bar{c}=1$. Analysing the second equation of Equation (A4) we consider two cases

- ${\bar{y}}_{a}=0$. Then from the third equation of Equation (A4) we get ${\bar{x}}_{a}=0$. Putting these results into the fourth equation of Equation (A4), with f(0)=ω, we obtain
  (A5) $$\begin{array}{c} -\alpha_{4}\omega \left(1-\gamma +\bar{z}\right)\bar{z}+\delta \left(\gamma -\bar{z}\right)=0\;. \end{array}$$
  Equation (A5) is a quadratic equation and has two solutions
  $$\begin{array}{cc} \; & {\bar{z}}_{1}=\frac{1}{2}\left(\gamma -1-\frac{\delta }{\alpha_{4}\omega }-\sqrt{{\left(1-\gamma +\frac{\delta }{\alpha_{4}\omega }\right)}^{2}+4\frac{\delta \gamma }{\alpha_{4}\omega }}\right),\\ \; & {\bar{z}}_{2}=\frac{1}{2}\left(\gamma -1-\frac{\delta }{\alpha_{4}\omega }+\sqrt{{\left(1-\gamma +\frac{\delta }{\alpha_{4}\omega }\right)}^{2}+4\frac{\delta \gamma }{\alpha_{4}\omega }}\right). \end{array}$$
  Solution ${\bar{z}}_{1}$ is negative because
  $$\begin{array}{cc} \gamma -1-\frac{\delta }{\alpha_{4}\omega }- & \sqrt{{\left(1-\gamma +\frac{\delta }{\alpha_{4}\omega }\right)}^{2}+4\frac{\delta \gamma }{\alpha_{4}\omega }}<\\ \; & <\gamma -1-\frac{\delta }{\alpha_{4}\omega }-|\gamma -1-\frac{\delta }{\alpha_{4}\omega }|\leq 0. \end{array}$$
  Solution ${\bar{z}}_{2}$ is positive because
  $$\begin{array}{cc} \gamma -1-\frac{\delta }{\alpha_{4}\omega }+ & \sqrt{{\left(1-\gamma +\frac{\delta }{\alpha_{4}\omega }\right)}^{2}+4\frac{\delta \gamma }{\alpha_{4}\omega }}>\\ \; & >\gamma -1-\frac{\delta }{\alpha_{4}\omega }+|\gamma -1-\frac{\delta }{\alpha_{4}\omega }|\geq 0. \end{array}$$
  We may note that ${\bar{z}}_{2}\leq \gamma$. Taking into consideration Equation (A1) we obtain the equilibrium point
  $$\left(\bar{x},{\bar{x}}_{a},{\bar{y}}_{a},\bar{z},\bar{c}\right)=\left(0,0,0,\frac{1}{2}\left(\gamma -1-\frac{\delta }{\alpha_{4}\omega }+\sqrt{{\left(1-\gamma +\frac{\delta }{\alpha_{4}\omega }\right)}^{2}+4\frac{\delta \gamma }{\alpha_{4}\omega }}\right),1\right)\;.$$
- ${\bar{y}}_{a}\neq 0$. From the second equation of Equation (A4) we get $1-\gamma +\bar{z}-{\bar{x}}_{a}-\bar{x}=0$. Substituting this to the fourth equation of Equation (A4) we obtain $\bar{z}=\gamma$. Now by $\bar{x}=0$ we have ${\bar{x}}_{a}=1$. Then from the third equation of Equation (A4) we obtain ${\bar{y}}_{a}=1$. We have the following equilibrium point
  $$\left(\bar{x},{\bar{x}}_{a},{\bar{y}}_{a},\bar{z},\bar{c}\right)=\left(0,1,1,\gamma ,1\right)\;.$$

□

Referring to the stability of the equilibrium points.

**Proposition** **A2.**
*The equilibrium points $S_{1}$ and $S_{2}$ are unstable and asymptotically stable, respectively.*

**Proof.** The Jacobi matrix *J* of Equation (A3) has the form

$$J=\left[\begin{array}{ccccc} -\alpha_{1}c & 0 & 0 & 0 & -\alpha_{1}x\\ -\alpha_{2}y_{a} & -\alpha_{2}y_{a} & \alpha_{2}\left(1-\gamma +z-x_{a}-x\right) & \alpha_{2}y_{a} & 0\\ 0 & \alpha_{3}\left(1-y_{a}\right) & -\alpha_{3}x_{a} & 0 & 0\\ \alpha_{4}f\left(x_{a}\right)z & A_{1} & 0 & A_{2} & 0\\ -\alpha_{1}c & 0 & 0 & 0 & -1-\alpha_{1}x \end{array}\right],$$

where

$$\begin{array}{cc} A_{1} & =-\alpha_{4}f^{\prime }\left(x_{a}\right)\left(1-\gamma +z-x_{a}-x\right)z+\alpha_{4}f\left(x_{a}\right)z,\\ A_{2} & =-\alpha_{4}f\left(x_{a}\right)\left(1-\gamma +z-x_{a}-x\right)-\alpha_{4}f\left(x_{a}\right)z-\delta . \end{array}$$

We study the stability of the equilibrium point $S_{1}$. For convenience we denote $S_{1}=\left(0,0,0,\bar{z},1\right)$, where $\bar{z}=\frac{1}{2}\left(\gamma -1-\frac{\delta }{\alpha_{4}\omega }+\sqrt{{\left(1-\gamma +\frac{\delta }{\alpha_{4}\omega }\right)}^{2}-4\frac{\delta \gamma }{\alpha_{4}\omega }}\right)$. The corresponding Jacobi matrix is

$$J\left(S_{1}\right)=\left[\begin{array}{ccccc} -\alpha_{1} & 0 & 0 & 0 & 0\\ 0 & 0 & \alpha_{2}\left(1-\gamma +\bar{z}\right) & 0 & 0\\ 0 & \alpha_{3} & 0 & 0 & 0\\ a_{4}\omega \bar{z} & \alpha_{4}\omega \bar{z} & 0 & -\alpha_{4}\omega \left(1-\gamma +2\bar{z}\right)-\delta & 0\\ -\alpha_{1} & 0 & 0 & 0 & -1\;, \end{array}\right]\;,$$

because $f\left(0\right)=\omega ,\;f^{\prime }\left(0\right)=0$. $J(S_{1})$ is a block matrix, so we have

(A6) $$\begin{array}{c} \det\left(J\left(S_{1}\right)-\lambda I\right)=\det\left(P_{1}-\lambda I\right)\cdot \det\left(P_{2}-\lambda I\right)\cdot \det\left(P_{3}-\lambda I\right), \end{array}$$

where

$$P_{1}=\left[-\alpha_{1}\right],$$

$$P_{2}=\left[\begin{array}{ccc} 0 & \alpha_{1}\left(1-\gamma +\bar{z}\right) & 0\\ \alpha_{3} & 0 & 0\\ \alpha_{4}\omega \bar{z} & 0 & -\alpha_{4}\omega \left(1-\gamma +2\bar{z}\right)-\delta \end{array}\right],$$

$$P_{3}=\left[-1\right].$$

The characteristic polynomial $w_{P_{2}}\left(\lambda \right)$ of matrix $P_{2}$ is

$$\begin{array}{cc} \; & w_{P_{2}}\left(\lambda \right)=\lambda^{2}\left(-\alpha_{4}\omega \left(1-\gamma +2\bar{z}\right)-\delta -\lambda \right)+\\ \; & +\left(\alpha_{4}\omega \left(1-\gamma +2\bar{z}\right)+\delta +\lambda \right)\alpha_{1}\alpha_{3}\left(1-\gamma +\bar{z}\right),\\ \; & w_{P_{2}}\left(0\right)=\alpha_{1}\alpha_{3}\left(1-\gamma +\bar{z}\right)\left(\alpha_{4}\omega \left(1-\gamma +2\bar{z}\right)+\delta \right). \end{array}$$

Now we examine the sign of $1-\gamma +\bar{z}$.

$$\begin{array}{cc} \; & 1-\gamma +\bar{z}=\frac{1}{2}\left(1-\gamma -\frac{\delta }{\alpha_{4}\omega }+\sqrt{{\left(\gamma -1+\frac{\delta }{\alpha_{4}\omega }\right)}^{2}+\frac{4\delta }{\alpha_{4}\omega }}\right)>\\ \; & >\frac{1}{2}\left(1-\gamma -\frac{\delta }{\alpha_{4}\omega }+|\gamma -1+\frac{\delta }{\alpha_{4}\omega }|\right)\geq 0, \end{array}$$

It follows that $1-\gamma +2\bar{z}>1-\gamma +\bar{z}>0$, then $w_{P_{2}}\left(0\right)>0$ and $w_{P_{2}}\left(\infty \right)=-\infty$. Therefore, there exits $\lambda_{0}>0$ such that $w_{P_{2}}\left(\lambda_{0}\right)=0$. The matrix $J(S_{1})$ has a positive eigenvalue; thus, the equilibrium point $S_{1}$ is unstable. Next we study the stability of the point $S_{2}$. The corresponding Jacobi matrix is

$$J\left(S_{2}\right)=\left[\begin{array}{ccccc} -\alpha_{1} & 0 & 0 & 0 & 0\\ -\alpha_{2} & -\alpha_{2} & 0 & \alpha_{2} & 0\\ 0 & 0 & -\alpha_{3} & 0 & 0\\ \alpha_{4}f\left(1\right)\gamma & \alpha_{4}f\left(1\right)\gamma & 0 & -\alpha_{4}f\left(1\right)\gamma -\delta & 0\\ -\alpha_{1} & 0 & 0 & 0 & -1 \end{array}\right].$$

$J(S_{2})$ is a block matrix; thus, we have

(A7) $$\begin{array}{c} \det\left(J\left(S_{1}\right)-\lambda I\right)=\det\left(R_{1}-\lambda I\right)\det\left(R_{2}-\lambda I\right)\det\left(R_{3}-\lambda I\right), \end{array}$$

where

$$R_{1}=\left[-\alpha_{1}\right],$$

$$R_{2}=\left[\begin{array}{ccc} -\alpha_{2} & 0 & \alpha_{2}\\ 0 & -\alpha_{3} & 0\\ \alpha_{4}f\left(1\right)\gamma & 0 & -\alpha_{4}f\left(1\right)\gamma -\delta \end{array}\right],$$

$$R_{3}=\left[-1\right].$$

The characteristic polynomial $w_{R_{2}}$ of matrix $R_{2}$ is:

$$w_{R_{2}}\left(\lambda \right)=-\lambda^{3}+v_{2}\lambda^{2}+v_{1}\lambda -\alpha_{2}\alpha_{3}\delta ,$$

where

$$\begin{array}{cc} v_{1} & =-(\alpha_{3}\alpha_{4}f\left(1\right)\gamma +\alpha_{2}\delta +\alpha_{3}\delta +\alpha_{2}\alpha_{3})<0,\\ v_{2} & =-(\alpha_{4}f\left(1\right)\gamma +\delta +\alpha_{2}+\alpha_{3})<0. \end{array}$$

Each coefficient of polynomial $w_{R_{2}}$ is negative, so each root has a negative real part. Matrices $R_{1}$ and $R_{3}$ have eigenvalues $-\alpha_{1}$ and −1, respectively, which are negative. Each eigenvalue of matrix $J(S_{2})$ has a negative real part, so the stationary state $S_{2}$ is asymptotically stable. □

We may note that from Equation (A3) we easily obtain the following remark.

**Remark** **A1.**

(a) *If $x_{a}\left(0\right)=0=y_{a}\left(0\right)$, then $\left(x\left(t\right),x_{a}\left(t\right),y_{a}\left(t\right),z\left(t\right),c\left(t\right)\right)\to S_{1}$ as t→∞.*
(b) *If $x_{a}\left(0\right)>0$ or $y_{a}\left(0\right)>0$, then $\left(x\left(t\right),x_{a}\left(t\right),y_{a}\left(t\right),z\left(t\right),c\left(t\right)\right)\to S_{2}$ as t→∞.*
